# Piloting a Postdischarge Virtual Glucose Monitoring Program for Patients With Diabetic Foot Wound in a Safety-Net Health System

**DOI:** 10.1177/19322968261469513

**Published:** 2026-07-21

**Authors:** Felona Gunawan, Blessing Johnny, Binsy Easow, Smitha Mathew, Jacqueline N. McNulty, Kristie Adame, Kaely Jackson, Larry S. Brown, Peter A. Crisologo, Uma Gunasekaran

**Affiliations:** 1 Division of Endocrinology and Metabolism, Department of Internal Medicine, University of Texas Southwestern Medical Center, Dallas, TX, USA; 2 Global Diabetes Program, Parkland Health, Dallas, TX, USA; 3 Office of Research Administration, Parkland Health, Dallas, TX, USA; 4 Department of Plastic Surgery and Division of Infectious Diseases and Geographic Medicine, Department of Internal Medicine, University of Texas Southwestern Medical Center, Dallas, TX, USA

**Keywords:** continuous glucose monitoring, diabetic foot wounds, postdischarge glucose monitoring, remote monitoring, safety-net health system

## Abstract

**Background:**

Diabetic foot wounds disproportionately affect patients from ethnic minorities and lower-socioeconomic status, many of whom face barriers to accessing diabetes technology. To evaluate whether short-term virtual glucose monitoring (VGM) has the potential to improve clinical outcomes in this high-risk population, we implemented a pilot VGM program within a safety-net health system.

**Method:**

We enrolled 40 hospitalized patients with diabetic foot wounds into a 3-month postdischarge VGM program that included 2 clinic visits and remote glucose monitoring every 1 to 2 weeks. Clinical outcomes were compared with a retrospective preintervention cohort of 78 similar patients.

**Results:**

Although both groups had similar HgbA1c at diagnosis (VGM 10.6 ± 1.8% vs preintervention 11.1 ± 2.0%, *P* = .20), the HgbA1c at 3 to 6 months was lower in the VGM cohort (7.6 ± 1.1% vs 8.5 ± 2.0%, *P* < .01). Wound healing occurred more frequently in the VGM participants, with 68% achieving wound closure by 3-4 months versus only 47% in the preintervention cohort (*P* = .04). Nonsignificant reductions in emergency department visits and hospital readmissions for wound complications or hypoglycemia were observed in the VGM versus the preintervention cohort. The VGM program allowed for more timely and frequent opportunities to adjust diabetes medications and address social barriers to care.

**Conclusions:**

A short-term postdischarge VGM program has the potential to not only increase access to diabetes technology but also meaningfully improve clinical outcomes among patients with diabetic foot wounds in a safety-net health setting. Such program may offer a scalable strategy to reduce rates of complications and lower healthcare costs in a safety-net health system.

## Introduction

Diabetic foot wound is the leading cause of nontraumatic lower extremity amputation (NLEA), and is more prevalent among individuals of ethnic minorities and lower-socioeconomic status.^1^ It imposes a substantial burden on both patients and the healthcare system, contributing to a high 30-day readmission rate of 20% to 30%, increased mortality, and an estimated annual treatment cost of $9 to $13 billion dollars in the United States.^2,3^ Approximately 30% to 40% of diabetic foot wounds heal within 12 weeks.^1,4^ Good glycemic control (HgbA1c <8% [64 mmol/mol]) is associated with lower risk of NLEA and may also lead to better wound healing outcomes.^5,6^

The use of continuous glucose monitoring (CGM) has increased among individuals with diabetes because of its many benefits, including lowering HgbA1c, reducing hypoglycemia, and increasing patient engagement in diabetes self-care practices.^7-10^ CGM also enables patients to share data remotely with their healthcare providers, which allows for the creation of virtual monitoring programs. However, CGM access remains limited for uninsured or under-insured patients in a safety-net setting.^11^ In this study, we piloted a short-term (3-month) postdischarge virtual glucose monitoring (VGM) program to determine whether such a program has the potential to improve glycemic control and foot wound healing outcomes in a safety-net health system.

## Methods

The study was approved by the University of Texas Southwestern Medical Center Institutional Review Board (STU 2021-1238) and the Parkland Office of Research Administration. The retrospective chart review for the preintervention cohort was exempt from informed consent requirement, while written informed consent was obtained from all participants of the VGM intervention cohort.

### Pre-Intervention Cohort

We reviewed electronic medical record of 78 adult patients (age ≥18 years) with diabetic foot wounds and HgbA1c >7% (53 mmol/mol) who had at least 1 visit at the diabetes clinic and the comprehensive wound care clinic at Parkland Health, which is the safety-net health system for Dallas County in Texas, between 2019 and 2022. Patients already using CGM were excluded. Data collected included demographics (age, sex, race/ethnicity, language, and insurance), diabetes type and complications, medications, HgbA1c, and wound measurements at the time of foot wound diagnosis and at 3 to 6 months after diagnosis, number of diabetes and wound clinic visits, and number of emergency department (ED) visits and/or hospital admissions within 3 to 4 months after diagnosis.

### Study (VGM) Cohort

We screened the inpatient podiatry and hospitalist service lists and enrolled 40 adults admitted with diabetic foot wounds with HgbA1c >7% (53 mmol/mol) between 2023 and 2025. Patients already using CGM were excluded. Participants were trained to use CGM 1 to 2 days prior to discharge. Each participant received 7 FreeStyle Libre 2 or 3 sensors (Abbott, Abbott Park, IL, USA), and the corresponding CGM reader (Abbott, Abbott Park, IL, USA) was provided only if their smartphone was incompatible with the Libre App. In person diabetes clinic visits occurred at 1 to 2 weeks and 3 months after discharge. Between the visits, an endocrinologist reviewed the CGM data remotely every 1-2 weeks and contacted patients only when needed for medication adjustments due to hyper- or hypoglycemia or missing CGM data. Only 1 patient was lost to follow-up and lacked a 3-month HgbA1c. Data collection mirrored that of the preintervention cohort, with the addition of CGM metrics and responses to a Social Determinants of Health (SDOH) questionnaire already embedded in the medical records addressing difficulty with paying for living expenses, food, housing, and transportation. Glycemic control and foot wound recurrence were also assessed at 1-year postintervention.

### Wound Assessment

Wound measurements were obtained from the clinical documentation of the comprehensive wound clinic providers, who were not informed of the patients’ study participation status. For wound healing time assessment, the time started with the first patient encounter with a wound clinic provider and ended on the encounter date when healed wound was documented by clinic providers. Wounds were considered healed if wound size of 0 × 0 × 0 cm (length, width, depth) or the phrase “wound healed” was documented on the encounter note. For the few VGM patients who were lost to follow-up from the wound clinic but came to the final diabetes clinic visit, wounds were considered healed if complete epithelialization over the wound area was visualized on exam. If the patient needed debridement or amputations after diagnosis of the original wound, the postsurgical wound measurements were used to determine wound healing outcomes.

### Statistical Analysis

Data were collected using Microsoft Excel (Microsoft Corporation, Redmond, WA, USA). Continuous variables were summarized as mean ± standard deviation and compared using *t* test. Categorical variables were summarized using proportions/percentages and compared using chi-square test. Pearson correlation coefficient was used to assess relationship between continuous variables. Multivariate linear regression was used to assess for potential confounding factors for continuous outcomes while multivariate logistic regression was used to assess for potential confounding factors for categorical outcomes. Analyses were performed using IBM SPSS Statistics (IBM, Armonk, NY, USA). A 2-tailed *P* value of <.05 was considered statistically significant.

## Results

**Figure 1.**
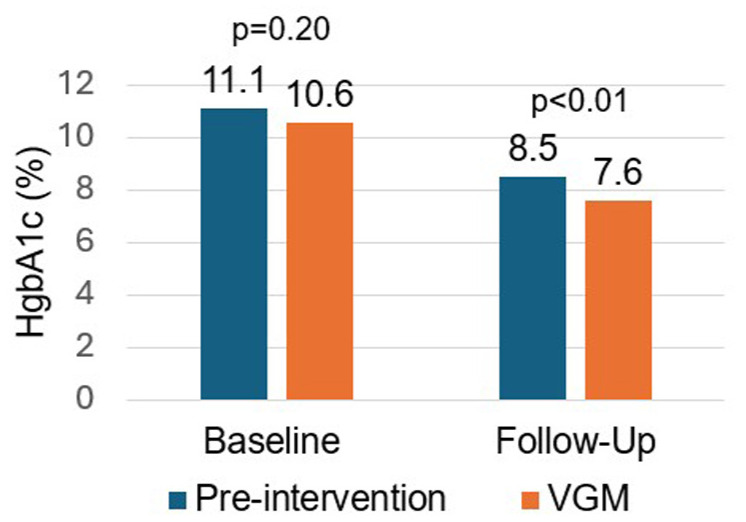
Although both cohorts had similar baseline HgbA1c, the VGM cohort had significantly lower HgbA1c at 3 to 6 months of follow-up compared with the preintervention cohort.

Baseline characteristics were similar between the preintervention and VGM cohorts, except for greater SGLT2 inhibitors (SGLT2i) use in the VGM cohort (Table 1). The majority of patients in both cohorts were Hispanic males with type 2 diabetes on insulin therapy receiving charity care. Baseline HgbA1c did not differ significantly (VGM 10.6 ± 1.8% [92 ± 19 mmol/mol] vs preintervention 11.1 ± 2.0% [98 ± 22 mmol/mol], *P* = .20). However, at 3 to 6 months, HgbA1c was significantly lower in the VGM compared with the preintervention cohort (7.6 ± 1.1% [60 ± 12 mmol/mol] vs 8.5 ± 2.0% [70 ± 22 mmol/mol], *P* < .01) (Figure 1). More patients in the VGM cohort (72.5%) had HgbA1c ≤8% compared with the preintervention cohort (41.0%) at 3 to 6 months. Among the VGM cohort, the average active CGM use was 69 ± 22%, with a glucose management indicator of 7.4 ± 0.8%, time in range of 61 ± 18%, and time below range of 1 ± 1% over 3 months. The time in range correlated well with the 3- to 6-month HgbA1c (*r* = –.74, *P* < .01) and was nonsignificantly higher (62% vs 57%, *P* = .4) in those with healed versus nonhealed wounds. There was no correlation between the average active CGM use and glycemic outcomes, but those with healed wounds had slightly higher average active CGM use (73% vs 60%, *P* = .08).

**Table 1.** Baseline Demographics of the Patients in the Preintervention and Study (VGM) Cohort.

	Preintervention cohort (n = 78)	Study (VGM) cohort (n = 40)	*P* value^a^
**Age** (years)	51 ± 9	50 ± 9	.33
**Male/female**, n (%)	63 (81%)/15 (19%)	28 (70%)/12 (30%)	.19
**Race/ethnicity**, n (%)			.23
Hispanic	53 (68%)	33 (82.5%)	
Black	16 (21%)	5 (12.5%)	
White/other	9 (11%)	2 (5%)	
**Language**, n (%)			.79
English	41 (53%)	20 (50%)	
Spanish	37 (47%)	20 (50%)	
**Insurance**, n (%)			.08
Charity	53 (68%)	34 (85%)	
Medicare/Medicaid	19 (24%)	3 (7.5%)	
Private	6 (8%)	3 (7.5%)	
**Type 2 diabetes**, n (%)	78 (100%)	38 (95%)	.11
**Baseline HgbA1c**, %	11.1 ± 2.0	10.6 ± 1.8	.20
**Complications/Comorbidities**, n (%)			
Retinopathy	59 (76%)	35 (88%)	.13
Albuminuria	59 (76%)	35 (88%)	.13
Hypertension	61 (78%)	32 (80%)	.82
Hyperlipidemia	71 (91%)	37 (93%)	1.00
Peripheral arterial disease	44 (56%)	25 (63%)	.53
**Medications**, n (%)			
Insulin	76 (97%)	38 (95%)	.60
SGLT2 inhibitor	8 (10%)	13 (33%)	<.01
GLP-1 receptor agonist	13 (17%)	9 (23%)	.44

a Mean ± standard deviation compared using *t* test. N (%) compared using chi-square test. A 2-tailed *P* value of <.05 was considered statistically significant.

**Figure 2.**
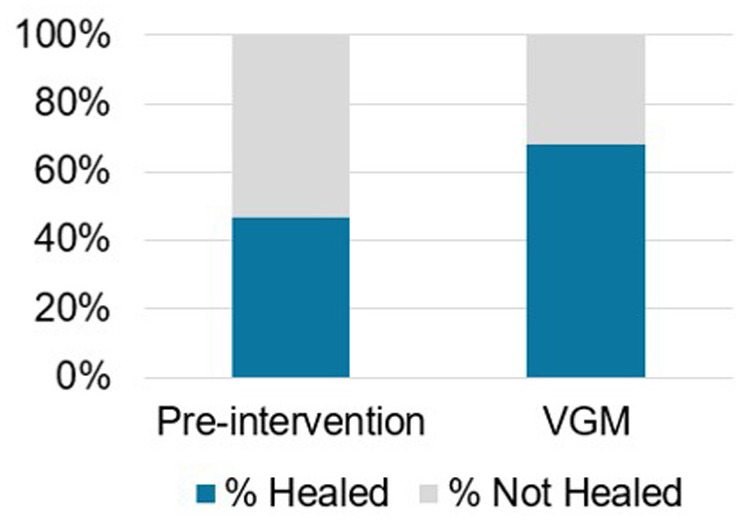
The VGM cohort had a higher proportion of patients with healed wounds by 3 to 4 months compared with the preintervention cohort (68% vs 47%, *P* = .04), despite similar prevalence of peripheral arterial disease (63% vs 56%, *P* = .53).

The VGM cohort had a higher proportion of patients with healed wounds by 3 to 4 months compared with the preintervention cohort (68% vs 47%, *P* = .04) (Figure 2), despite similar prevalence of peripheral arterial disease (63% vs 56%, *P* = .53). Among patients who achieved wound healing, the average time to documented wound healing was similar between groups (VGM 73 days vs preintervention 63 days, *P* = .30). Nonsignificant reductions in the percentage of patients with ED visits and hospital admissions for diabetic foot wounds (18% vs 24%, *P* = .40) and hypoglycemia (0% vs 3%, *P* = .55) were observed in the VGM compared with the preintervention cohorts within 3 to 4 months.

Implementation of the CGM virtual monitoring program increased the average number of diabetes clinic visits from 1.3 to 2.0 (*P* < .05) within 3 to 4 months after foot wound diagnosis and decreased the median wait time to the first diabetes clinic visit from 59 to 11 days (*P* < .05). The average number of successfully completed phone calls was 4 within 3 months. Although most patients found CGM helpful and wished to continue using it, only 25% were able to do so due to insurance coverage or ability to pay out of pocket. Most patients reported significant socioeconomic challenges, including difficulty paying for living expenses (73%) and housing (30%), food insecurity (38%), and lack of transportation (25%).

For the 38 VGM cohort patients who have follow-up data 1-year postintervention, 19 patients (50%) were able to maintain their HgbA1c ≤8%, including the 7 patients who continued CGM use for >3 months after the study. In addition, there were slightly less patients in the HgbA1c ≤8% group (12/19, 63%) compared with the HgbA1c >8% group (15/19, 79%) who either had diabetic foot wound recurrence or never healed their wounds by 1-year follow-up.

## Discussion

Consistent with prior reports, we found that diabetic foot wounds were more prevalent among patients of ethnic minority groups, who also faced significant SDOH challenges.^12,13^ This pattern is expected, as these populations often experience inconsistent access to healthcare and frequently present with advanced diabetes complications. Compounding this disparity, these same patients typically have limited access to diabetes technologies such as CGM,^11^ despite evidence that CGM improves glycemic control and may reduce diabetes-related complications.^7-10^ In this study, we piloted a short-term VGM program using CGM for patients with diabetic foot wounds to evaluate its potential impact on clinical outcomes and to assess whether such program warrants long-term investment by the healthcare system.

Participation in the VGM program was associated with improved glycemic control within 3 to 6 months, which is likely due to several reasons. By design, the program enabled more timely and frequent contact with diabetes clinic providers, facilitating faster medication titration. It also created more opportunities to address SDOH-related barriers. For example, during the weekly phone calls, we identified challenges related to insulin cost and transportation and were able to intervene before the patients’ next clinic visit. The remote nature of the program further mitigated transportation barriers. Moreover, as previously reported, use of real-time CGM can help patients understand how different factors, especially diet, affected their glucose levels, which in turn motivated lifestyle changes and improved engagement in diabetes self-management.^10^ Because of the limitations of our study design, it was not possible to pinpoint which of these factors (CGM use versus more frequent provider contact versus addressing SDOH concerns) contributed most to improvement in glycemic control.

Notably, there was a higher percentage of patients who were on SGLT2i therapy at baseline in the VGM compared with the preintervention cohort (33% vs 10%) due to increased access to SGLT2i over the study period. However, there was no significant difference in baseline HgbA1c between the 2 cohorts and insulin titration was the primary diabetes therapy modification during the VGM program. Moreover, using a multivariable linear regression, we confirmed that SGLT2i use was not a confounder of the relationship between the VGM program and 3- to 6-month-HgbA1c (coefficient 0.33 [95% confidence interval [CI] –0.55, 1.21], *P* = .45).

Although good glycemic control has been associated with lower risk of NLEA, its relationship with wound healing remains less certain.^5,6^ In our study, a higher percentage of patients with healed wounds within 3 to 4 months was observed in the VGM versus the preintervention cohort. However, factors beyond glycemic control, such as increased patient engagement with care and enhanced support for navigating SDOH challenges, likely contributed to this outcome. The slightly higher (albeit nonsignificant) average active CGM use indicates possible increased engagement in those patients with healed wounds. No other CGM metrics correlated with wound healing outcome. Use of SGLT2i at baseline, which was higher in the VGM cohort, was not a confounder in the relationship between the VGM program and whether patients healed their wound as assessed using multivariate logistic regression (coefficient 0.86 [95% CI 0.31, 2.37], *P* = .77). Greater use of SGTL2i in the VGM cohort at baseline also did not appear to negatively impact wound healing outcomes despite earlier concerns that SGLT2i use, particularly canagliflozin, increased NLEA risk.^14^ The association between SGLT2i use and NLEA risk was also not observed in subsequent large clinical trials.^15,16^

Despite strong interest in continuing CGM use after participation in the VGM program, most patients were unable to do so due to cost barriers, and subsequently a significant portion of the patients had worsening of their HgbA1c to >8% at 1 year follow-up. Given the evidence suggesting greater benefit with continuous rather than intermittent CGM use, long-term CGM access would likely be advantageous for patients with diabetic foot wounds who are at high risk for diabetic foot wound recurrence and hospital readmissions.^1,17,18^ Although not statistically significant due to the low number of occurrences, we observed lower ED visits and readmissions rates for diabetic foot wound complications and hypoglycemia in the VGM cohort versus the preintervention cohort. The average cost of CGM was approximately $500 per patient for the 3-month period, which if extended to an entire year supply would cost approximately $2000 per patient. Integrating a virtual monitoring program within our diabetes clinic did not incur significant additional clinic operation costs since we were already routinely conducting virtual visits, using CGM platforms, and we were able to employ our existing workforce consisting of endocrinologists, advanced practice providers, and certified diabetes care and education specialists. Considering that the average cost of admission for NLEA ranges from $40 000 to $70 000 and for hypoglycemia $11 000 to $17 000,^18,19^ extending the VGM program to a longer duration for patients with diabetic foot wounds may offer meaningful opportunities for healthcare cost savings if it could successfully improve clinical outcomes and reduce rates of acute care use.

Limitations of this study include the pre- and postintervention cohort design rather than a randomized controlled trial, which limits our ability to confidently attribute the observed outcomes to the intervention implemented. There was also a large time gap between the preintervention and the VGM cohorts, which was mostly due to unexpected delay in obtaining the CGM for the study. This introduces other confounding factors such as differences in standard of care, medication availability, COVID-associated healthcare disruptions, and evolving institutional practices, which could independently account for the outcome differences. However, being in a safety-net setting does restrict our ability to rapidly integrate changes in standard of care guidelines, especially with regards to access to new medications and technology, and most of the patients in both cohorts were primarily managed with insulin therapy. In addition, we did not collect comprehensive data on diabetic foot wound factors, such as peripheral arterial disease severity, history of prior amputation, revascularization, offloading, antibiotic use, specific surgical intervention, which could have affected wound healing outcome. The ED visit and/or readmission occurrences were also not high enough to detect statistically significant differences in the 2 cohorts given the small sample size and short duration of follow-up. To better assess the potential impact of the VGM program for patients with diabetic foot wounds and expand the generalizability of the study findings, a larger (preferentially multicentered) randomized controlled trial of longer duration is needed.

## Conclusions

In conclusion, a short-term virtual monitoring program using CGM has the potential to increase access to diabetes technology, improve glycemic control, and enhance wound healing outcomes for patients with diabetic foot wounds within a safety-net health system. Such a program, if proven to be effective in a larger randomized clinical trial, may offer a scalable strategy to reduce rates of complications and lower healthcare costs in a safety-net health system.
